# The adamant adherence to a prior belief: the case of anosognosia in anorexia nervosa

**DOI:** 10.3389/fneur.2025.1670485

**Published:** 2025-09-05

**Authors:** Stelina Rushani, Gerardo Salvato, Manuela Sellitto

**Affiliations:** ^1^Department of Brain and Behavioral Sciences, University of Pavia, Pavia, Italy; ^2^Cognitive Neuropsychology Centre, ASST “Grande Ospedale Metropolitano” Niguarda, Milan, Italy; ^3^NeuroMi, Milan Center for Neuroscience, Milan, Italy

**Keywords:** anorexia nervosa, anosognosia, body image, dopaminergic reward system, insula, interoception, multisensory integration, prediction error

## Abstract

Anorexia Nervosa (AN) is a complex psychiatric disorder marked by restrictive eating and distorted body image. Often, individuals with AN show a persistent denial of illness severity, even in the presence of their pathology’s life-threatening consequences. This condition, known as anosognosia, has been extensively investigated in right-brain damaged patients who deny their contralesional motor deficit (anosognosia for hemiplegia; AHP). In the present perspective, we draw parallels with AHP in the attempt to explain anosognosia for the illness in AN through the most recent theoretical computational accounts. We review evidence suggesting that anosognosia in AN may be rooted in unbalanced prediction error and suboptimal multisensory integration mechanisms. Specifically, individuals with AN would normally rely more heavily on exteroceptive information at the expense of signals coming from the inside of the body—i.e., interoception—, in addition to which a distorted body memory overrides new incoming sensory information, leading to inadequate updated body image. These disrupted processes potentially involve dysfunctional insular, striatal, and prefrontal regions, whereby alterations in the dopaminergic reward system may reinforce maladaptive behaviors and attenuate responses to updating feedback. We propose a neurocognitive model according to which individuals with AN would rely excessively on outdated third-person body representations, failing to integrate new egocentric sensory cues. This perspective may offer ideas for future applications in cognitive rehabilitation of AN.

## 1 Introduction

A complex psychiatric pathology that profoundly affects behavior, thoughts, and relationship with one’s own body is anorexia nervosa (AN). AN is an eating disorder that most commonly affects adolescent girls and is characterized by persistent food restriction, an intense fear of gaining weight, and a significantly distorted body perception and representation (DSM-5) (1). This symptomatology can heavily influence everyday life behavior to the point that, for instance, individuals with AN tend to walk through door-like openings as if their bodies were larger than they actually are (2). Remarkably, this distortion persists even when they are asked to only imagine the action rather than to physically perform it (3). In addition, individuals with AN often underestimate their interoceptive and metacognitive body-related abilities on heartbeat counting or body size estimation tasks, even when being provided with positive external feedback after a successful performance or with evidence by the clinician regarding their actual body size (4). Importantly, a high percentage of individuals with AN denies there is anything wrong in the early months or years of their illness, and clinical observations reveal that they tend to have difficulties updating their persuasion of “being fat” (5). This display of denial over the gravity of the illness in individuals with AN affects extremely their compliance with therapy (5, 6).

Despite their different clinical presentations—*in primis*, the absence of a focal brain lesion—, the lack of awareness of being ill in AN, accompanied by altered body image distortions, resembles the symptomatology described in the anosognosia for hemiplegia (AHP). AHP is a neurological condition in which patients with right-brain damage deny their deficit and claim that their contralesional paralyzed limbs are functioning normally (7–13). Even when presented with robust external evidence (e.g., inability to clap their hands), these patients remain unaware of their impairment (14) and are more likely to claim their plegic hand moved after self-initiated actions rather than passive movements, suggesting that motor intention (high order function) predominate over sensory feedback (15). However, despite their verbal or behavioral denial, studies show that, often, AHP patients’ implicit awareness about the deficit is preserved (16–19). For instance, in coordinated bimanual tasks (e.g., opening a jar), they can initially attempt the action as if both limbs were functioning, but subsequently adjust their movements by using the intact limb only, indicating subconscious recognition of the paralysis (20). AHP is recently increasingly explored through predictive coding frameworks that emphasize the *discrepancy between expected and actual bodily states*. This frame of reference, which we briefly review below, might as well account for anosognosia for the illness in AN.

In AHP, comparator models suggest that one evaluates the match between predicted motor outputs and actual sensory information (“affordances”). As a result, AHP patients might code the representations of the predicted consequences of having made the movement rather than the actual experience of (non) movement in the present (21). Both motor control and motor awareness activate motor and premotor brain regions, suggesting the existence of overlapping neural pathways: although motor areas are impaired, there might still be some preserved premotor activation, and this residual pattern of activation might support the false belief of being able to move, thereby preventing the proper monitoring of such failed movements (22, 23). Evidence of preserved motor intentionality and planning for the plegic hand in AHP comes from the bimanual coupling effect, where the plegic hand influences the intact hand, as in healthy controls, while this constraint is absent in non-anosognosic hemiplegic patients (15, 23). Furthermore, activations in the cortical regions responsible for motor control, preceding the false experience of movement, suggest that this comparator system might misidentify the actual consequences of the missed movement of the paralyzed limb (16). Damage to the comparator system may cause more severe motor awareness dysfunction in AHP than in conditions such as motor neglect, where performance improves after external feedback (23–25).

On this ground, computational neuroscience models conceive AHP as a result of a disrupted process in which the agents’ prior beliefs (top-down), deriving from predictive internal models on how they see the world based on past experience (being able to move), antagonize the actual input from the ascending exteroceptive (i.e., coming from outside the body) and interoceptive (i.e., coming from the internal body) signals (bottom-up).

The mismatch between expectations and actual sensory inputs (i.e., unpredicted outcomes or omissions) would normally generate a so-called prediction error (16). Prediction error is a phasic dopaminergic response occurring in the midbrain, striatum, prefrontal cortex, and associated structures, and it reflects the difference in value between a predicted outcome (in this context, the intended movement/action of the limb) and the outcome that actually occurred (26). Lesions associated with AHP seem to disrupt frontostriatal dopaminergic pathways, corroborating the notion that a compromised prediction error process, and subsequent impaired learning, would be responsible for the deficit in awareness for hemiplegia (16). Moreover, also alteration in metacognitive abilities might account for the lack of recognition of actual body condition. For example, AHP may not only entail a deficit in drawing new inferences based on retrospective (i.e., from memory) beliefs of their motor abilities, but also a deficit in evaluating prospective (i.e., future) motor abilities (27, 28). Inconsistent forms of awareness may stem from a failure to transfer information from working memory to long-term memory: patients might deny being paralyzed while agreeing to use a wheelchair or remain in bed (29).

Initial predictive models from computational neuroscience propose that distorted body image perception in individuals with AN might be rooted in prior beliefs of body memories that are not up to date based on current sensory information (i.e., multisensory dis-integration) (30). While little is known about the shared neural processes between AHP and the lack of awareness for the illness in AN, the observations of altered frontostriatal and insular responses during unexpected rewards or omissions (31, 32) suggest that the dopaminergic reward system is a potential target for further investigation not only in AHP but also to explain anosognosia in AN (14, 16, 33). In this perspective, we aim to outline a theoretical predictive coding framework for anosognosia for the illness in AN, drawing on current evidence of altered multisensory integration and abnormal prediction error to propose a neurocognitive model to account for this disturbance.

## 2 Understanding anosognosia in anorexia nervosa through multisensory dis-integration

To have a coherently integrated bodily experience and, thus, a coherent bodily self-awareness we must combine the information that comes from external sources (e.g., visual, somatosensory, auditory signals) with internal bodily signals (e.g., interoceptive, vestibular, proprioceptive signals) in a so-called optimal multisensory integration (16, 34, 35), thus considering information from the two spatial frames of reference: egocentric (first-person perspective) and allocentric (third-person perspective) (36). Against this background, internal bodily signals could be degraded or down-weighted in AHP, contributing to the false experience of movement (16), as well as AHP patients may experience an illusory sense of being able to move their impaired limb in the first-person perspective, without experiencing the same phenomenon when someone else moves the limb passively for them (27). Similarly, individuals with AN seem to engage in obsessive mirror checking and treat the body as an object (also known as “self-objectification”), which may reflect a tendency to internalize a mirror perspective (third-person/allocentric), rather than a self-perspective (first-person/egocentric). For instance, they are more accurate than controls in judging a hand’s spatial orientation, suggesting that they do not suffer from biomechanical constraints (i.e., physical or mechanical limitations imposed by the body structure), but from an overreliance on visual rather than experiential internal strategies (37). Moreover, also evidence from a classic paradigm, the Rubber Hand Illusion, points at altered multisensory integration in AN, although with conflicting results (38). For example, individuals with eating disorders show higher proprioceptive drift and higher embodiment scores as compared with healthy controls, indicating a stronger influence of visual input and, thus, increased plasticity of the bodily self (39). Conversely, other studies report no general group differences, but rather a disassociation between implicit and explicit domains, with implicit visuo-motor measures revealing stronger visual capture of proprioception (40), often associated with lower implicit body satisfaction linked to perceptual experience (41). However, AN is not merely a consequence of a maladaptive overdependence on visual cues; the complex multisensory integration deficit extends beyond vision as a primary source of reliance, including a disrupted integration of other signals into a coherent body image. Among these are tactile signals, indicated by the overestimation of tactile distances together with diminished interoceptive accuracy during a blindfolded distance estimation task (42). However, to maintain a coherent long-term mental representation of our body image, we must integrate multisensory information with body memories. According to the “allocentric lock theory,” individuals with AN would be stuck in a pre-existent memory of a larger body image and fail to update this allocentric body representation with egocentric information, for instance, through interoceptive signals (37, 43–47). Thus, it is possible that the false beliefs about body size in AN might result from biased priors (i.e., old images of one’s own body stored in memory) that are not correctly updated by incoming sensory information (36, 48). As an example, in a virtual body illusion task, prior erroneous beliefs/memories about the body (i.e., overestimation of a virtual body) were associated with greater posterior bodily estimation distortions (i.e., overestimation of one’s own body after the illusion) (30). Thus, individuals with AN would probably lock their perception into a distorted prior belief that subsequently influences the posterior estimation of bodily state (30). Additionally, greater deficits in multisensory integration have a positive correlation with greater disorder symptomatology (36, 49), therefore, this dysregulated updating mechanism might explain why the more severe the anorectic condition, the more the affected individuals tend to “deny” their illness (8). Of note, often individuals with AN seem to have explicit knowledge of their condition, but they do not appropriately experience it, namely, they behave as if they were not ill. This also implies that people with AN constantly put their body in situations of danger, such as severe malnutrition (50, 51). This seems quite the opposite of what can be observed in some patients with AHP who, when explicitly asked, deny their condition but implicitly behave as if they knew about it (52).

## 3 Dopaminergic reward system dysfunctions might sustain anosognosia in anorexia nervosa

Potential dysfunctions of the dopaminergic reward system have been proposed as a trait marker of AN, with abnormalities persisting even after weight restoration (53). In healthy participants, a prediction error computed by comparing the current bodily state (e.g., being hungry to respond to the biological need for food) and the anticipated one (e.g., feeling satiated) would normally elicit an approach reaction toward food cues. However, this same prediction error would induce a learned aversive response in AN, where avoiding food is necessary to obtain the long-term reward of becoming thinner (54) and the rewarding value of consuming food and feeling satiated is coded as punishment (55). Neurally, a pattern of connectivity from the ventral striatum towards the hypothalamus, evidenced in AN and in the opposite direction than what occurs in healthy hunger regulation, is indeed typically associated with fear response (32, 33). Moreover, the amygdala, which processes the emotional valence related to food cues, would influence episodic memories stored in the hippocampal complex by instating strong biased priors (56): this can further influence prediction error by physiologically adapting the body to starvation and suppressing appetite-inducing signals, thus overly preserving the illness (57). Such starvation and suppressed appetite would mean blunted bodily reactions to feeding-regulation cues (50).

Even though individuals with AN may acknowledge the negative consequences of their behaviors, they appear not to be able to properly code incoming bodily states (8, 35). As a consequence, they may fail to appropriately recognize the body’s physiological condition based to internal cues (i.e., interoceptive abilities), maintaining a persistent desire for thinness despite severe weight loss. This long-term reward and the lack of insight about the actual physical condition, thus, possibly reflect a maladaptive reinforcement mechanism (16, 48). In this vein, the weight loss phase in AN would be itself perceived as a reward where the person subsequently increases tolerance to slimming, similar to mechanisms of drug addiction, with denial as one of its core features (8). This increased capacity to delay long-term gratification (i.e., enhanced self-control) in AN extends beyond food-related cues (58), and is alike to what has been noted in patients with lesions to the insular cortex who showed not only reduced sensitivity to immediate gratification, but also blunted arousal when facing cues with positive valence (59). Consistent evidence, indeed, has long indicated that the insular cortex is a region crucial for both the processing and the integration of interoceptive signals into conscious feelings as well as for goal-oriented decision-making (60). In particular, the insula would play an important role in encoding prediction error by acting as an anticipatory signaler (33, 61, 62), initiating the so-called “as if body loop” chain of physiologic events. Specifically, when making a decision, the insula would simulate in the present the somatosensory state associated with a future event, thus guiding behavior [i.e., “somatic marker hypothesis”; (63)]. A recent account indicates that AN would be sustained by a deficit in the circuit that connects subcortical (striatum and amygdala) and cortical areas (frontal, somatosensory, and parietal) (64): although these areas may not be impaired in AN per se, the insula, as a connection hub, would be dysfunctional, thus leading to a disrupted communication between them. This idea is corroborated by the evidence of functional anomalies in the right insula along with associated parietal areas, commonly observed in AN (65). Therefore, it is possible that a dysfunctional activity in the insula prevents a correct body representation, particularly deficit in interoceptive awareness: this would make insular dysfunction a potential underlying cause of the persistent belief of being overweight in AN, with difficulty in updating with current new interoceptive information (66, 67).

## 4 A neurocognitive model explaining anosognosia in anorexia nervosa

AN is a severe psychiatric condition characterized by extreme weight loss, rigid control of eating behavior, and body image disturbance. One of its peculiar clinical features is *a persistent lack of insight about the condition and its severity*: despite evident extreme low body weight and medical complications, people with AN often deny being ill (7). They may express concern about secondary symptoms (i.e., vomiting, fatigue, gastrointestinal pain), yet they consistently fail to attribute these symptoms to the eating disorder, thereby focusing on the consequences of malnutrition itself rather than recognizing their restrictive behavior (68).

To interpret this phenomenon, we attempt to propose a neurocognitive model of anosognosia for the illness in AN that draws from computational theoretical accounts of AHP, namely, theories that try to explain the behavior of patients who deny their motor deficit even when confronted with clear contrary evidence (16). Of note, not only the lack of awareness of being ill in AN cannot be attributed to a focal brain lesion, but this symptom also manifests differently. While patients with AHP explicitly deny their inability but often behave as if they implicitly knew about their deficit, individuals with anosognosia for AN often seem to have declarative knowledge of their condition but they do not appropriately experience it, thus behaving as if they were not ill (69). From this perspective, the individual with AN’s experience of “feeling fat” should be interpreted as a persistent belief of being overweight (i.e., prior), according to which individuals with AN enforce maladaptive behaviors for weight loss. This prior would be so biased that, when compared with new incoming sensory information (i.e., the likelihood: exteroceptive, interoceptive, and proprioceptive information that should signal that the body is in fact underweight), would result in an unbalanced prediction error and, thus, in the inability to update correctly the body image (i.e., posterior). At the core of our model, however, an abnormal prediction error mechanism also builds on disrupted multisensory integration. Anosognosia in AN would be nourished by the inability to appropriately evaluate and update one’s own body shape from an egocentric perspective that grounds on interoceptive and proprioceptive input. Indeed, such information seems to be down-weighted in favor of visual (exteroceptive) information (47, 70). This overreliance on visual stimuli would shape a persistent allocentric perspective of the body (43) and this imbalance would contribute to the inability to correctly update the body representation in the presence of conflicting information (i.e., being extremely thin as indicated by internal bodily signals) (48). Individuals with AN seem to consider their body as an external object (e.g., as suggested by excessive mirror checking) (37) as if they did not have the “competence” that healthy individuals show when changes occur in/to their body (i.e., interoceptive awareness). This phenomenon resembles, in its clinical manifestations, to AHP, where patients fail to update their belief of motor functioning, even in the presence of sensory evidence of the paralysis (16). These dysfunctional multisensory integration and prediction error mechanisms would impair bodily awareness by creating a fracture between the “past” body (i.e., before the disease), seen from an allocentric perspective (i.e., body memory), and the “new” body (i.e., as indicated by interoceptive, exteroceptive, and proprioceptive information) (49). This would prevent a veridical comparison between outdated body memory information with the new incoming one. In a similar way, patients with AHP would remain anchored to a previous “memory” of a moving limb despite it is now severely plegic.

**Figure 1:**
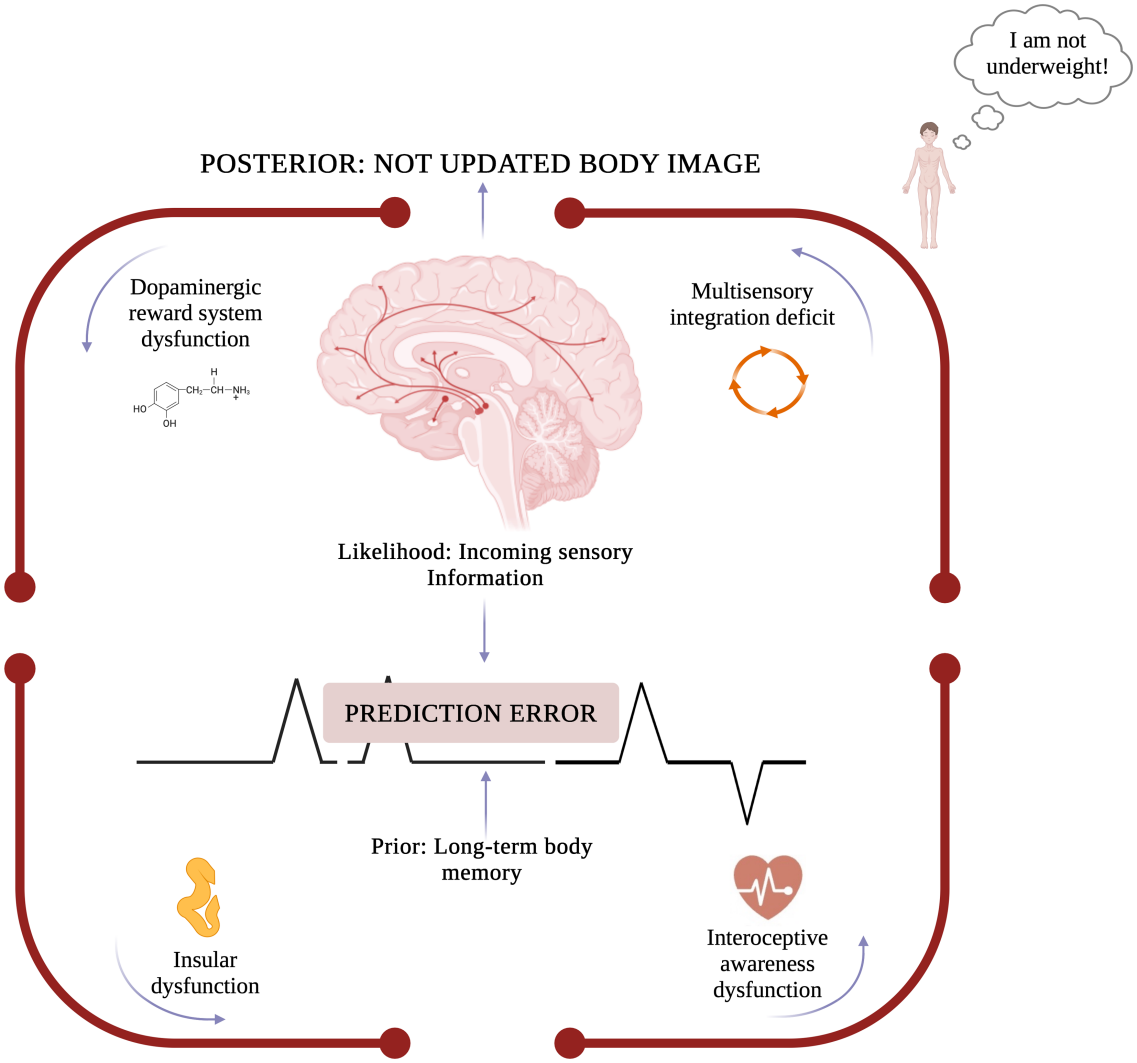
Neurocognitive mechanisms involved in anosognosia for anorexia nervosa. The model illustrates how anosognosia in anorexia nervosa arises from abnormal prediction error signaling, where long-term body memory (prior) overrides incoming sensory information (likelihood), preventing body image updating (posterior). This imbalance is nourished by multisensory integration deficits (i.e., visual overreliance and down-weighted internal bodily signals), based on insular dysfunctions and dopaminergic system alterations, leading to the distorted belief “I am not underweight!”. Created in BioRender. Rushani S. (2025) https://BioRender.com/9qqf73q.

At the basis of prediction error dysregulation is the dopaminergic reward system, which contributes both to the onset and the maintenance of AN (32, 33, 53). Unlike typical reward learning, where actual physiological feelings, such as hunger, are prioritized, individuals with AN exhibit a higher sensitivity to long-term goals (i.e., being thin). This dysfunctional pattern persists even after weight restoration, where AN individuals continue to restrict food intake, further compromising the prediction error processing. As a result, the body would not be able to distinguish correctly between “I am full” and the actual bodily state “I am starving.” At the neuroanatomical level, this impaired prediction error processing, and thus the diminished interoceptive response to bodily needs, is likely underpinned by neural abnormalities in a key region, namely the insula (71). In the case of AN, a possible explanation could be a dysregulation of the insula as a crucial hub between cortical and subcortical areas, which further compromises sensitivity to internal cues (such as hunger state) (50, 54). As such, the down-weighting of interoceptive signals, such as hunger and satiety further compromise the generation of accurate prediction errors. This failure to recognize bodily states crystalizes maladaptive prior beliefs and would contribute to the false experience of body efficiency despite physiological starvation, *ultimately supporting the denial of illness in individuals with AN* (Figure 1).

Budling on the proposed framework, and in addition to pharmacological treatments (e.g., haloperidol or olanzapine) that have been tested for treating delusional, obsessive thinking about the body and to restore weight in AN (72, 73), our model supports interventions targeting specific neurocognitive mechanisms. Specifically, it encourages the use of interoceptive training (74, 75), reward-based training (76, 77), as well as therapies focused on multisensory integration by using virtual reality (78–80) and body illusion paradigms (81). Moreover, therapeutic approaches that have been used for AHP, specifically those based on error-awareness detection (82), could also be explored within the context of treating anosognosia in AN. Thus, our model highlights the integration of non-pharmacological treatments that target neurocognitive mechanisms to foster profound, long-term changes, while reducing the burden of side effects often associated with medication.

## 5 Conclusion

Anorexia nervosa (AN) is a multifaceted psychiatric disorder marked by a profound and persistent misperception of body image. A key feature of AN is anosognosia for the illness. We propose a neurocognitive model that explains this phenomenon through the interplay of two interrelated mechanisms: impaired prediction error processing and disrupted multisensory integration. Specifically, our model situates anosognosia in AN within a predictive coding account, emphasizing how disrupted belief updating and abnormal weighting of sensory information jointly sustain denial of illness. Together, these disturbances hinder accurate updating of body image, which remains fixed on an overweight representation, despite contradictory evidence. By drawing from computational accounts used to explain anosognosia for hemiplegia (AHP), we extend a well-established neurocognitive framework to the psychiatric domain, offering a transdiagnostic perspective on impaired awareness across conditions. In this sense, with the present model we aim to move from descriptive accounts of denial of illness in AN to a more mechanistic explanation that links clinical symptoms to specific neurocognitive processes. Fundamental mechanisms implicated in the lack of awareness for motor deficits in AHP—such as disrupted interoception processing and unbalanced reward-based prediction error—are paralleled in AN, suggesting shared vulnerabilities across conditions. As a result, rigid mental representations prove highly resistant to clinical intervention. Considering the impact that denial has on treatment resistance, advancing our understanding of the neurocognitive underpinnings of anosognosia in AN may provide crucial insights for treatment, paving the way for novel approaches to cognitive rehabilitation, especially targeting interoceptive awareness.

## Data Availability

The original contributions presented in the study are included in the article/supplementary material, further inquiries can be directed to the corresponding author.
